# FRAILTY AND THE COMPARATIVE EFFECTIVENESS AND SAFETY OF SGLT2I AND DPP4I IN OLDER ADULTS WITH TYPE 2 DIABETES

**DOI:** 10.1093/geroni/igz038.2157

**Published:** 2019-11-08

**Authors:** Dae H Kim, Ajinkya Pawar, Seoyoung Kim, Elisabetta Patorno

**Affiliations:** 1 Hebrew SeniorLife, Boston, Massachusetts, United States; 2 Brigham and Women’s hospital, Boston, Massachusetts, United States

## Abstract

We conducted a 1:1 propensity score-matched retrospective cohort study of 83,432 patients with type 2 diabetes (mean age, 71.5 years [standard deviation, 5.0]) initiating a SGLT2i or a DPP4i in Medicare data. We estimated HRs (95% CIs) for a composite cardiovascular endpoint and severe hypoglycemia comparing the two treatments in the entire population and by the CFI-based frailty subgroups. Compared with DPP4i, SGLT2i were associated with a lower rate of the composite cardiovascular endpoint (HR, 0.70 [95% CI, 0.64-0.77]) and a similar rate of severe hypoglycemia (0.88 [0.71-1.07]) over a mean follow-up of 8.8 months. The rate of composite cardiovascular endpoint for SGLT2i vs DPP4i was consistently lower in pre-frail (0.71 [0.64-0.79]) and frail (0.67 [0.55-0.80]) subjects, but not in non-frail patients (0.98 [0.62-1.54]). The rate of severe hypoglycemia was not meaningfully different between SGLT2i and DPP4i (non-frail, 0.39 [0.12-1.16]; pre-frail, 0.83 [0.65-1.07]; frail, 1.13 [0.78-1.64]).

